# Luteolin Isolated from the Flowers of *Lonicera japonica* Suppresses Inflammatory Mediator Release by Blocking NF-κB and MAPKs Activation Pathways in HMC-1 Cells

**DOI:** 10.3390/molecules15010385

**Published:** 2010-01-18

**Authors:** Ok-Hwa Kang, Jang-Gi Choi, John-Hwa Lee, Dong-Yeul Kwon

**Affiliations:** 1 Department of Oriental Pharmacy, College of Pharmacy, Wonkwang University, Wonkwang Oriental Medicines Research Institute, Jeonbuk 570-749, Korea; E-Mails: kangokhwa@daum.net (O-H.K.); jj0038@wku.ac.kr (J-G.C.); 2 College of Veterinary Medicine and Bio-Safety Research Institute, Chonbuk National University, Jeonju, 561-756, Korea; E-Mail: johnhlee@chonbuk.ac.kr (J-H.L.)

**Keywords:** *Lonicera japonica*, pro-inflammatory cytokine, intracellular Ca^2+^, NF-κB, MAPKs

## Abstract

Luteolin (3′,4′,5,7-tetrahydroxylflavone) is a plant flavonoid and pharmacologically active agent that has been isolated from several plant species. In the present study, the effect of luteolin from the flowers of *Lonicera japonica* on phorbol 12-myristate 13-acetate (PMA) plus A23187-induced mast cell activation was examined. Luteolin significantly inhibited the induction of inflammatory cytokines such as tumor necrosis factor (TNF)-α, interleukin (IL)-8, IL-6 and granulocyte-macrophage colony-stimulating factor (GM-CSF) by PMA plus A23187. Moreover, luteolin attenuated cyclooxygenase (COX)-2 expression and intracellular Ca^2+^ levels. In activated HMC-1 cells, the phosphorylation of extra-signal response kinase (ERK 1/2) and c-jun *N*-terminal Kinase (JNK 1/2), but not p38 mitogen-activated protein kinase (p38 MAPK) were decreased by treatment of the cells with luteolin. Luteolin inhibited PMA plus A23187-induced nuclear factor (NF)-κB activation, IκB degradation, and luciferase activity. Furthermore, luteolin suppressed the expression of TNF-α, IL-8, IL-6, GM-CSF, and COX-2 through a decrease in the intracellular Ca^2+^ levels, and also showed a suppression of the ERK 1/2, JNK 1/2, and NF-κB activation. These results indicated that luteolin from the flowers of *Lonicera japonica* exerted a regulatory effect on mast cell-mediated inflammatory diseases, such as RA, allergy disease and IBD.

## 1. Introduction

Mast cells are one of the major effecter cells in the immune response system. Activated mast cells release pro-inflammatory cytokines, such as TNF-α, IL-6, IL-8, IL-13, GM-CSF, and inflammatory mediators including histamine, leukotrienes, serotonin, prostaglandin (PG)E_2_ as well as PGD_2_ [1,2,3]. Cytokines, such as IL-6, TNF-α, IL-8 and GM-CSF, are released in a coordinated network and play an important role in chronic inflammation. As such, the pattern of cytokine expression largely determines the nature and persistence of the inflammatory response [4]. TNF-α is preformed and stored in granules of mast cells or newly synthesized following mast cell activation; it is a multifunctional cytokine and an important mediator of the immune and inflammatory response. TNF-α is an autocrine stimulator as well as a potent inducer of other inflammatory cytokines, including IL-1β, IL-6, IL-8 and GM-CSF [5,6]. IL-6 is a pro-inflammatory cytokine and a potent mediator of inflammatory processes [7]. GM-CSF also plays an important role in the development and perpetuation of inflammation [8]. A previous study reported that histamine induced GM-CSF production in human bronchial epithelial cells [9]. Cytokines produce their cellular effects by activation of various transcription factors such as AP-1 and NF-κB. Furthermore, the expression of many of these cytokines and their receptors are upregulated by these transcription factors. 

Ca^2+^ acts as a second messenger during cell activation. An increase in the levels of intracellular Ca^2+^ has been proposed to act as an essential trigger for mast cell activation [10,11]. It has also been reported that the release of intracellular Ca^2+^ from internal stores is required for MAPK activation [12]. Recently, studies showed the involvement of MAPK and NF-κB activation by Ca^2+^, with increased Ca^2+^ levels inducing the release of biological mediators including TNF-α, IL-6 and IL-8 [13,14]. Moreover, NF-κB activation was reported to be required for the expression of many inflammatory proteins such as GM-CSF, TNF-α, IL-6, COX-2 and inducible nitric oxide synthase (iNOS) [15]. Therefore, inhibition of NF-κB could reduce the expression of inflammatory genes and is a mechanism by which anti-inflammatory agents might elicit their anti-inflammatory effects [16].

*Lonicera japonica* Thunb.(Caprifoliaceae) is known in traditional Korean medicine as an anti-inflammatory treatment. It has a strong scent typical of an aromatic medicinal plant, and its flowers are used to treat skin inflammations and wounds. *Lonicera japonica* was found to have anti-tumor effects in human lung carcinoma cells [17], and anti-allergic effects [18]. Flavonoids such as luteolin are ubiquitous plant secondary metabolites and have a variety of biological effects, including anti-inflammatory and anti-allergic properties; some of these compounds are also known to inhibit the release of histamine from mast cells [19]. Luteolin was shown to inhibit pro-inflammatory cytokine production *in vitro* [20], and suppresses inflammation-associated gene expression by blocking the NF-κB and AP-1 activation pathway [21]. These reports suggest that the anti-inflammatory effect of luteolin isolated from the flowers of *Lonicera japonica* may be through potent inhibition of mast cell activation. However, no preexisting study has been reported on mast cell-mediated anti-inflammatory activity of luteolin isolated from the flowers of *Lonicera japonica*. Thus, as a part of our ongoing screening program to evaluate the anti-inflammatory potential of natural compounds, we investigated the in *vitro* anti-inflammatory activity of *Lonicera japonica* through activity-guided fractionation. Subsequently, the effects of luteolin isolated from the flowers of *Lonicera japonica* were evaluated on PMA plus A23187-induced pro-inflammatory mediators by inhibiting MAPKs and IκBα/NF-κB signal pathways in HMC-1 cells. 

## 2. Results and Discussion

Many recent studies on plant-derived anti-inflammatory compounds have investigated the potential inhibitory effects of natural products using *in vivo* and *in vitro* systems. Luteolin, as a plant flavonoid, is an active oriental medicine ingredient that has been isolated from several plant species and used since ancient times to cure diseases such as inflammation, allergy and cancer. However, no report has been issued on the anti-inflammatory effects of luteolin isolated from the flowers of *Lonicera japonica* or on the mode of action of its active constituents.

**Figure 1:**
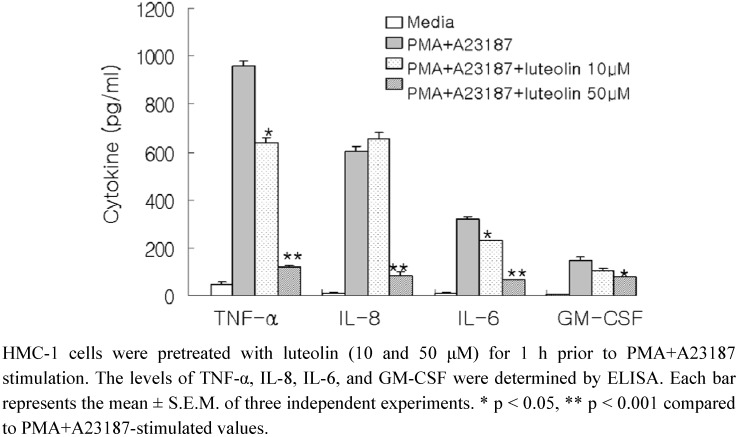
Effect of luteolin on production of pro-inflammatory cytokines in PMA plus A23187-induced HMC-1 cells.

In the present study, luteolin isolated from the flowers of *Lonicera japonica* was investigated for mast cell-mediated anti-inflammatory effects. To evaluate the potential effects of luteolin on the production of pro-inflammatory cytokines, we pretreated the cells with luteolin (10 and 50 μM) before stimulation with PMA (50 nM) and A23187 (1 μM) for 8 h, and further analysis using ELISA. As shown in Figure 1, the levels of TNF-α, IL-8, IL-6 and GM-CSF were considerably increased after stimulation with PMA plus A23187 in HMC-1. Pretreatment of cells with luteolin (10 and 50 μM) significantly inhibited the increase of these protein levels in a concentration-dependent manner. The maximal inhibition of TNF-α, IL-8, IL-6 and GM-CSF production by luteolin (50 μM) was approximately 87%, 86%, 78%, and 46%, respectively. Moreover, we examined the cytotoxicity of luteolin on HMC-1 cells using the MTT assay. Luteolin did not exhibit any cytotoxic effects up to 100 μM (data not shown). 

**Figure 2:**
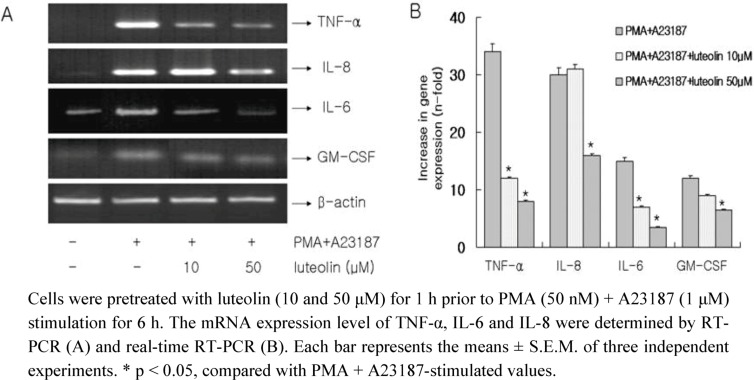
Effect of luteolin on gene expression of pro-inflammatory cytokines in PMA plus A23187-induced HMC-1 cells.

Next, the pro-inflammatory cytokine gene expression was then analyzed using RT-PCR and real-time RT-PCR (Figure 2). Enhanced TNF-α, IL-8, IL-6 and GM-CSF mRNA expression induced by PMA plus A23187 was inhibited by pretreatment of cells with luteolin at a concentration of 50 μM. Pretreatment with 10 μM of luteolin only slightly decreased the gene expression of TNF-α and IL-6, but not the other cytokines.

**Figure 3:**
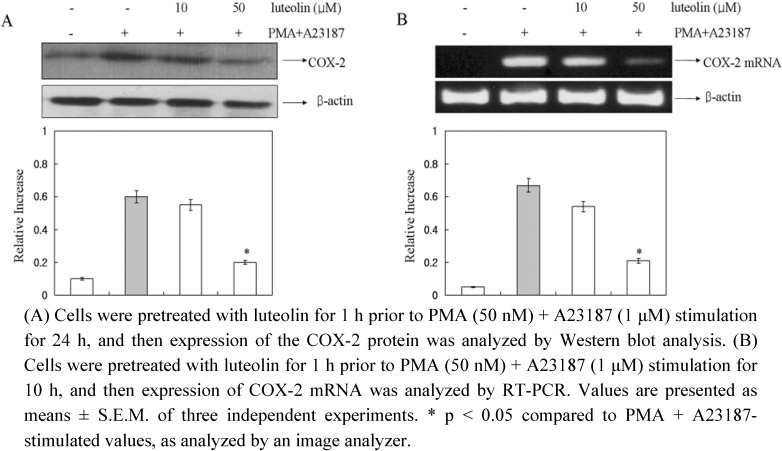
Effect of luteolin on COX-2 protein and mRNA expression in PMA plus A23187-induced HMC-1 cells.

In a recent study, it was demonstrated that COX-2 played important roles in mast cell-mediated inflammation [22]. So, to determine the effect of luteolin on COX-2 protein and COX-2 mRNA expression induced by PMA plus A23187, we performed Western blot and RT-PCR analysis. The cells were pretreated with luteolin (10 and 50 μM) for 1 h and then treated with PMA plus A23187 for 10 h. As shown in Figure 3, luteolin inhibited the PMA plus A23187-induced expression of COX-2 protein and mRNA levels.

**Figure 4:**
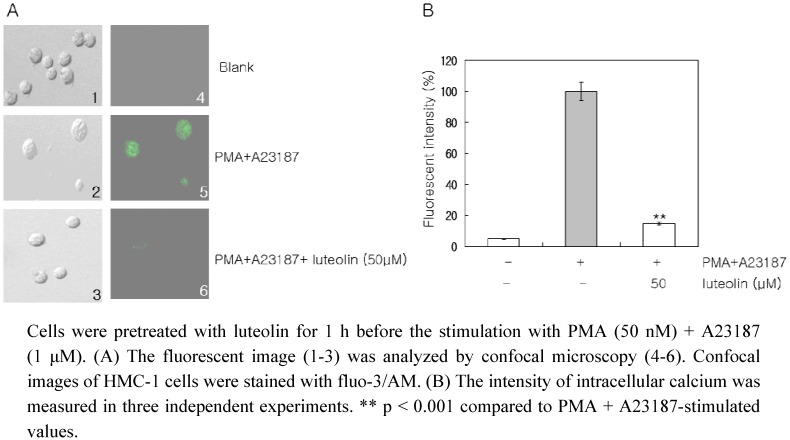
Effect of luteolin on intracellular calcium levels.

Calcium acts as a secondary messenger during cell activation [10]. An increase in the intracellular Ca^2+^ levels has been proposed to be an essential trigger for mast cell activation [23]. An increased intracellular Ca^2+^ level induces the release of biological mediators including TNF-α, IL-8 and IL-6 [24]. It has also been reported that the release of intracellular Ca^2+^ from internal stores is required for MAPK activation [12]. A depletion of intracellular Ca^2+^ inhibited pro-inflammatory cytokine expression *via* NF-κB signaling pathway in RBL-2H3 cells [14]. From the results of the present study, we suggest that the inhibitory effects of luteolin on the expression of TNF-α, IL-8, IL-6 and GM-CSF levels is mediated by the reduction of intracellular Ca^2+^ in HMC-1 cells. We also investigated the effect of luteolin on the intracellular levels of Ca^2+^, using confocal laser microscopy to detect the fluorescence signal coming from individual cells. The PMA plus A23187 treatment considerably increased the intracellular Ca^2+^ levels, but pretreatment of the cells with luteolin (50 μM) inhibited this increase in intracellular Ca^2+^ levels (Figure 4).

**Figure 5:**
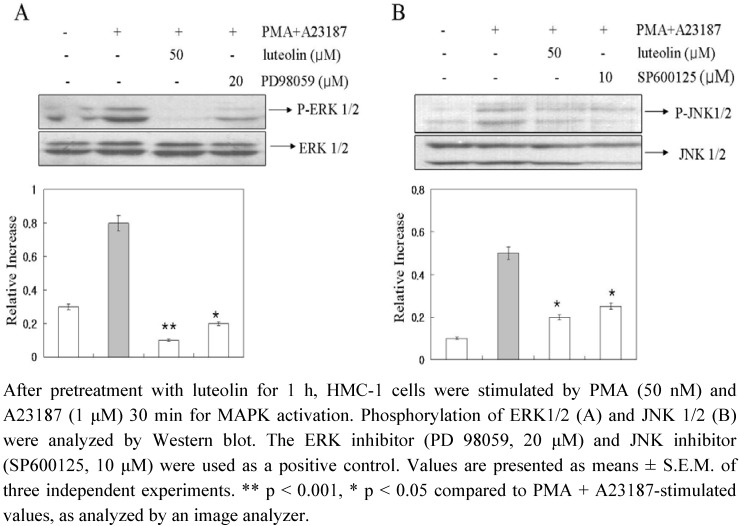
Effect of luteolin on activation of MAPKs.

In an attempt to evaluate the mechanisms underlying the effects of luteolin, we examined the potential effects of luteolin on activation of MAPKs. The stimulation of HMC-1 cells with PMA plus A23187 resulted in an increased phosphorylation of all three types of MAPKs, p38, JNK, and ERK, after 15–30 min (data not shown). As shown in Figure 5, luteolin attenuated PMA plus A23187-induced phosphorylation of ERK 1/2 and JNK 1/2, but did not affect the phosphorylation of p38 MAPK (data not shown). The present study showed that luteolin inhibited the phosphorylation of ERK and JNK but not of p38 MAPK (Figure 5). Furthermore, luteolin demonstrated a greater level of inhibition of ERK and JNK-phosphorylation than the ERK 1/2 inhibitor (PD98059) and JNK 1/2 inhibitor (SP600125). These data suggested that luteolin inhibited pro-inflammatory cytokine production and intracellular Ca^2+^ release *via* inhibition of ERK and JNK activation. 

**Figure 6:**
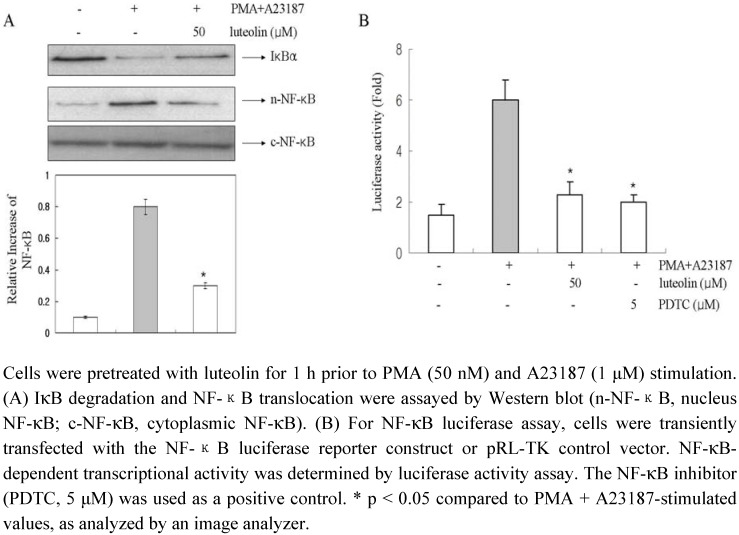
Effect of luteolin on the activation of NF-κB in HMC-1 cells.

To evaluate the mechanism by which luteolin affected the gene expression of pro-inflammatory cytokines, we examined the effects of luteolin on NF-κB activation. The expression of the pro-inflammatory cytokines tested in this study is known to be regulated by a transcription factor, NF-κB [25]. Stimulation of HMC-1 cells with PMA plus A23187 induced the degradation of IκBα and promoted the nuclear translocation of p65 NF-κB after 2 h of incubation (Figure 6A). Luteolin inhibited the PMA plus A23187-induced degradation of IκBα as well as the nuclear translocation of p65 NF-κB. 

In order to confirm these results, we examined the possible effects of luteolin on the NF-κB dependent gene reporter assay (Figure 6B). We transiently transfected HMC-1 cells with the pNF-κB luciferase reporter vector and pRL-TK vector and incubated the transfected cells with PMA plus A23187 in the presence or absence of luteolin. As shown in Figure 6B, the PMA plus A23187 treatment increased the reporter gene expression but this increased activity was significantly decreased by treatment with luteolin (50 μM). The PDTC (NF-κB inhibitor) used as a positive control. This implies that luteolin might inhibit COX-2 expression through suppression of NF-κB activation in HMC-1.

Mast cells contain potent mediators, including histamine, heparin, proteinases, leukotrienes, and multifunctional cytokines; these molecules potentially contribute to the inflammatory processes and also play an important role [26]. Mast cell-derived pro-inflammatory cytokines, particularly TNF-α, IL-8, IL-6 and GM-CSF have a critical biological role in allergic inflammation. In this study, we demonstrated that luteolin suppressed the expression of TNF-α, IL-8, IL-6 and GM-CSF in PMA plus A23187-induced HMC-1 cells. These data indicated the anti-inflammatory effect of luteolin as a potent inhibitor of mast cell activation. Also, we tested the effects of luteolin on COX-2 expression and MAPK phosphorylation together with intracellular Ca^2+^ release in HMC-1 cells. The results indicated that luteolin reduced pro-inflammatory cytokine production, COX-2 expression and intracellular Ca2+ release *via* the inhibition of MAPK activation.

Recently, a number of published studies have indicated the interference of luteolin with NF-κB [21]. Because this transcription factor is strongly linked to inflammatory and immune responses, we postulate that luteolin mediates its effects at least partly through suppression of NF-κB activation. Although NF-κB activation is regulated by MAPKs through multiple mechanisms, accumulating evidence indicates that NF-κB activation is modulated by MAPKs that induce site-specific phosphorylation of an inhibitory protein called IκB. Activation of NF-κB is dependent on the degradation of IκBα, an endogenous inhibitor that binds to NF-κB in the cytoplasm [27]. So, the role of NF-κB activation is important for the regulation of cytokine production in inflammatory condition. Expression of the TNF-α, IL-6, IL-8 and GM-CSF genes are dependent on the activation of transcription factor NF-κB in mast cells. This implies that luteolin might inhibit the expression of inflammatory mediators through the suppression of NF-κB activation and IκB degradation in HMC-1. Luteolin was shown to decrease the degradation of IκBα and nuclear translocation of p65 NF-κB in PMA plus A23187-stimulated mast cells. This demonstrated that luteolin inhibited the PMA and A23187-induced TNF-α, IL-6, IL-8, GM-CSF and COX-2 expression through a decrease in the intracellular levels of Ca^2+^, ERK 1/2, JNK 1/2, as well as activation of NF-κB.

## 3. Experimental

### 3.1. Reagents

PMA, the calcium ionophore A23187 (calcymycin; C_29_H_37_N_3_O_6_), PD98059, SP600125, SB203580 and 3-[4,5-dimetylthiazol-2-yl]-2,5-diphenyltetrazolium bromide (MTT) were purchased from the Sigma Chemical Co. (St. Louis, MO, USA). Iscove's modified Dulbecco's medium (IMDM) was obtained from Gibco BRL (Grand Island, NY, USA); anti-human TNF-α, IL-6, IL-8, and GM-CSF antibodies, the biotinylated anti-human TNF-α, IL-6, IL-8, and GM-CSF antibodies, and recombinant human TNF-α, IL-6, IL-8, and GM-CSF were purchased from BD PharMingen (San Diego, CA, USA); the NF-κB, and IκB antibodies from Santa Cruz Biotechnology (Santa Cruz, CA, USA); Lipofectamine™ 2000 was obtained from Invitrogen (Carlsbad, CA, USA), the NF-κB Luciferase Reporter vector from Panomics Inc; and the Dual-Luciferase® Reporter Assay System from Promega (Madison, WI, USA). SYBR Premix Ex TaqTM was purchased from Takara Bio (Shiga, Japan).

### 3.2. Plant materials

The flowers of *Lonicera japonica* purchased from the Daehak Hanyak kuk Oriental drug store (Iksan, Korea), were authenticated by Dr. D.Y. Kwon. A voucher specimen (No.08-030) was deposited in the Laboratory of Herbology, College of Pharmacy, Wonkwang University, Iksan, Korea. 

### 3.3. Isolation of luteolin

The MeOH extracts were partitioned with organic solvents of different polarities to yield *n*-hexane, EtOAc, *n*-BuOH and H_2_O fractions, in sequence. The EtOAc fraction of each plant was subjected to silica gel chromatography with CH_2_Cl_2_-MeOH (lower layers, by volume, 30:1→1:1, 100% MeOH, gradient) as the solvents to yield the *Lonicera japonica* luteolin. The structure of the compound was determined by its physico-chemical and spectral data (LC-MS, 1D and 2D NMR) which were in agreement with those reported in the literature [28,29].

### 3.4. Cell culture

The HMC-1 cells were grown in IMDM and supplemented with 100 U/mL of penicillin, 100 μg/mL of streptomycin, and 10% fetal bovine serum (FBS) at 37 °C in 5% CO_2_ with 95% humidity. The HMC-1 cells were treated with luteolin (10 μM to 50 μM) for 1 h. The cells were then stimulated with 50 nM of PMA plus 1 μM of A23187 and incubated at 37 °C for the indicated time periods.

### 3.5. MTT assay

For the MTT colorimetric assay of cell survival, we used a method described by Kang *et al.* [30]. Cell aliquots were seeded (3 × 10^5^) in microplate wells and incubated with 20 μL of an MTT solution (5 mg/mL) for 4 h at 37 °C under 5% CO_2_ and 95% air. This was followed by the addition of 250 μL of DMSO to extract the MTT formazan. An automatic microplate reader was used to read the absorbance of each well at 540 nm.

### 3.6. Cytokine assay

The HMC-1 cells were pretreated with various concentrations of luteolin (10 to 50 μM) for 1 h before PMA plus A23187-stimulation. We then used the enzyme-linked immunosorbent assay (ELISA) method to assay the culture supernatants for the TNF-α, IL-8, IL-6 and GM-CSF protein levels. To measure the cytokines, we used a modified ELISA method by Kang *et al*. [31]. 

### 3.7. RNA isolation, reverse transcription (RT) analysis

Using a GeneAll^R^ RiboEx RNA extraction kit (GeneAll Biotechnology, Republic of Korea), we isolated total RNA from HMC-1 cells in accordance with the manufacturer's specifications. The concentration of total RNA in the final eluate was determined by spectrophotometry. The total RNA (2.0 μg) was heated at 65 °C for 10 min and then cooled on ice. A cDNA synthesis kit (iNtRON Biotech, Republic of Korea) was used for 90 min at 37 °C to reverse-transcribe each sample to cDNA. Primer sequences for TNF-α, IL-6, IL-8, GM-CSF, COX-2 and β-actin were used for PCR analysis as described previously [32]. The PCR products increased as the concentration of RNA increased. Finally, the products were electrophoretically resolved on a 2.0% agarose gel and visualized by staining with ethidium bromide.

### 3.8. Real-time RT-PCR

The levels of TNF-α, IL-6, IL-8, GM-CSF and β-actin mRNA were measured with the real-time reverse transcription (RT)-PCR method using SYBR green. Total RNA was extracted from the cells with an RNeasy^®^ Mini kit (Qiagen Inc., Valencia, CA, USA). Aliquots (1 μg) of total RNA were used for RT, using a PrimeScriptTM RT reagent kit (Takara Bio, Shiga, Japan) and a Smart cycler^®^ II System Takara Bio, Shiga, Japan). The RT reaction was performed in total volume was 20 μL using a SYBR Premix Ex TaqTM (Takara Bio); 2 μL of cDNA sample was used as a template. Their sequences are shown in Table 1. Cycling was started with an activation step at 95 °C for 10 s, and amplification program was repeated 45 times (denaturation, 95 °C for 5 s; annealing/extension, 60 °C for 20 s) with fluorescence measurement at 72 °C.

The fluorescence of the SYBR green dye was determined as a function of the PCR cycle number. In order to confirm amplification specificity, the PCR products from each primer pair were subjected to a melting curve analysis. The ΔCT values (C_t_= cycle threshold value) for the housekeeping gene (β-actin) and the target gene (TNF-α, IL-6, IL-8 and GM-CSF) were calculated by subtracting the experiment group (PMA+A23187+luteolin) from the control (nonstimulated value). The relative expression of the target gene was calculated on the basis of 2^-∆ (∆Ct)^. The ∆ (∆Ct) values were calculated by subtracting the drug treated (PMA+ A23187+ luteolin) ∆Ct from the control (PMA+ A23187) ∆Ct. The primer sequences for target genes are described in Table 1.

**Table 1:** Sequences of oligonucleotide primers designed for real-time PCR.

	Forward (5’–3’ orientation)	Reverse (5’–3’ orientation)	Accession no.
hTNF-α	GACAAGCCTGTAGCCCATGTTGTA	CAGCCTTGGCCCTTGAAGA	NM_ 000594.2
hIL-6	AAGCCAGAGCTGTGCAGATGAGTA	TGTCCTGCAGCCACTGGTTC	NM _000600.1
hIL-8	ACACTGCGCCAACACAGAAATTA	TTTGCTTGAAGTTTCACTGGCATC	NM_ 00584.2
hGM-CSF	ACCATGATGGCCAGCCACTAC	GTGATAATCTGGGTTGCACAGGAA	NM_000758
β-actin	ATTGCCGACAGGATGCAGAAG	ATGGAGCCACCGATCCACA	NM_ 0016142

The primers pairs were designed using Primer Express^®^ software.

### 3.9. Fluorescent measurements of the intracellular Ca^2+^ level

The intracellular Ca^2+^ values were obtained from a single cell using Fluo-3/AM, the fluorescent Ca^2+^-sensitive indicator. The cells were incubated with 4 μM Fluo-3/AM at 37 °C for 30 min, and then washed with PBS. After addition of the culture medium, the temperature was maintained at 37 °C for 10 min, and then the cells were viewed using confocal laser scanning microscope (Olympus, Japan). The Fluo-3 loaded cells were illuminated with the 488 nm line of an argon laser and the emitted fluorescence was collected through a 20× water-immersion objective and by setting the confocal pinhole to 2 μM. The intensity of fluorescence was detected using one of two photomultipliers. To obtain a good spatial image, three successive frames were collected for each cell. The intracellular Ca^2+^ level was evaluated by its fluorescent intensity [33].

### 3.10. Preparation of cytoplasmic and nuclear extracts

Nuclear and cytoplasmic extracts were prepared as described elsewhere [30]. Briefly, after activating the cells for the time periods indicated, 5 × 10^6^ cells were washed with ice-cold PBS and centrifuged at 15,000 × g for 1 min. The cells were then resuspended in 40 μL of a cold hypotonic buffer (10 mM Hepes/KOH, 2 mM MgCl_2_, 0.1 mM EDTA, 10 mM KCl, 1 mM DTT, and 0.5 mM PMSF, pH 7.9). The cells were allowed to swell on ice for 15 min after which they were lysed gently with 2.5 μL of 10% Nonidet P (NP)-40. The lysate was centrifuged at 15,000 × g for 3 min at 4 °C. The supernatant was collected and used as the cytoplasmic extract. The nuclear pellets were gently resuspended in 40 μL of cold saline buffer (50 mM HEPES/KOH, 50 mM KCl, 300 mM NaCl, 0.1 mM EDTA, 10% glycerol, 1 mM DTT, and 0.5 mM PMSF, pH 7.9) and left for 20 min on ice. After centrifuging at 15,000 × g for 15 min at 4 °C aliquots of the supernatent containing nuclear proteins were frozen in liquid nitrogen and stored at − 70 °C until further analysis. The bicinchoninic acid protein assay (Sigma, St. Louis, MO, USA) was used for protein quantitation.

### 3.11. Western blot analysis

HMC-1 cells (5 × 10^6^ cells/well) were stimulated with PMA (50 nM) plus A23187 (1 μM). Cell lysates were then prepared in a sample buffer containing sodium dodecyl sulfate (SDS). The samples were heated at 95 °C for 5 min and briefly cooled on ice. Following a centrifugation step at 15,000 × g for 5 min, the proteins in the cell lysates were separated by 10% SDS-polyacrylamide gel electrophoresis (SDS-PAGE) and transferred to a nitrocellulose membrane. The membrane was then blocked with 5% skim milk in PBS-Tween-20 for 1 h at room temperature and then incubated with anti-NF-κB, and IκB. After washing the blot in PBS-Tween-20 three times, the blot was incubated with a secondary antibody for 1 h and then antibody-specific proteins were visualized using an enhanced chemiluminescence detection system in accordance with the recommended procedure (Amersham Corp., Newark, NJ, USA). The each protein levels were quantities with an image analyzer (FC-26WL, Vilber Lourmat, Marne-La-Vallee, France).

### 3.12. Transient transfection and a luciferase assay

For the transfection, HMC-1 cells were seeded (1 × 10^7^) in a 100 mm culture dish. Lipofectamine™ 2000 (Invitrogen, Carlsbad, CA, USA) was used to transiently transfect pNF-κB luciferase reporter vector and pRL-TK control vector constructs into HMC-1 cells. In brief, the cells were incubated at 37 °C in a 5% CO_2_ incubator for 48 h, and then, the transfected HMC-1 cells were plated and stimulated with PMA plus A23187. Luteolin was added 1 h before stimulation. Four hours after stimulation, the cells were harvested and washed in cold PBS before lysing in a 500 μL lysis buffer (Dual-Luciferase® Reporter Assay System; Promega). After vortex mixing and centrifugation at 12,000 × g for 3 min at 4 °C, the supernatant was stored at −70 °C until further analysis. For the luciferase assay, 20 μL of cell extract was mixed with 100 μL of the luciferase assay reagent at room temperature. To measure the luciferase activity, a luminometer (1420 luminescence counter, Perkin Elmer) was used in accordance with the manufacturer's protocol. All the transfection experiments were performed independently at least three times, showing similar results. The relative luciferase activity was defined as the ratio of firefly luciferase activity to renilla luciferase activity. 

### 3.13. Statistical analysis

Statistical significances were compared between each treated group and analyzed by the Student’s *t*-test. The data from the experiments are presented as means ± S.E.M. The numbers of independent experiments assessed are given in the figure legends. 

## 4. Conclusions

Luteolin regulated the production of TNF-α, IL-6, IL-8 and GM-CSF in PMA plus A23187-stimulated HMC-1 cells. Luteolin also decreased COX-2 expression and intracellular Ca^2+^ release. Furthermore, luteolin inhibited the ERK 1/2, JNK 1/2, and NF-κB pathway. Therefore, the regulation of the NF-κB signal pathway by luteolin isolated from the flowers of *Lonicera japonica* in HMC-1 cells is a potentially attractive and characteristic probe for studying mast cell-mediated inflammatory diseases, such as IBD, RA, and allergy disease. 
